# PERSPECTIVES OF PERSON-CENTEREDNESS ACROSS LONG-TERM CARE AND COMMUNITY-BASED SETTINGS

**DOI:** 10.1093/geroni/igae098.0678

**Published:** 2024-12-31

**Authors:** Lauren Stratton, Sheryl Zimmerman, Lea Efird-Green, Sam Fazio

**Affiliations:** Alzheimer’s Association, Chicago, Illinois, United States; University of North Carolina at Chapel Hill, Chapel Hill, North Carolina, United States; University of North Carolina at Chapel Hill, Chapel Hill, North Carolina, United States; Alzheimer’s Association, Chicago, Illinois, United States

## Abstract

Within long-term care, person-centeredness is key to providing quality care to people living with dementia. However, there are discrepancies on how long-term and community-based organizations provide care that is person-centered and how it is integrated within their organizations and among staff. To better understand the concept of person-centeredness within long-term and community-based services, we conducted three focus groups with members of the Alzheimer’s Association Dementia Care Provider Roundtable, which consists of leadership of long-term and community-based care organizations. Prompts asked questions on person-centeredness in relation to current organizational systems, care, and workforce. Overall, themes emerged on types of care that are delivered in a person-centered way, challenges in delivering care in a person-centered care, the integration of person-centeredness into staff training, and if person-centered is the correct term to use. Additionally, discussion included integrating technology in a more person-centered way to benefit both the staff and the person living with dementia. Understanding these themes may help to provide recommendations on how to create guidelines for training staff and integrate person-centered care with the long-term care workforce more effectively.

